# Métastase musculaire d'un carcinome épidermoide du col utérin: à propos d'un cas avec revue de la littérature

**DOI:** 10.11604/pamj.2014.18.23.4442

**Published:** 2014-05-07

**Authors:** Imane Mezouri, Hanane Chenna, Sara Bellefqih, Hanan ElKacemi, Tayeb Kebdani, Noureddine Benjaafar

**Affiliations:** 1 Service de Radiothérapie, Institut National de l'Oncologie, Université Mohammed V Souissi, Rabat, Maroc

**Keywords:** Carcinome épidermoïde, muscle, col utérin, métastase, cancer, squamous cell carcinoma, muscle, cervix, metastasis, cancer

## Abstract

Les métastases musculaires sont rares, leur nombre est probablement sous estimé. Nous rapportons le cas d'une patiente âgée de 65 ans, ayant présenté une métastase musculaire d'un carcinome épidermoïde du col utérin 6 mois après le diagnostic de la tumeur primitive. Le diagnostic a été obtenu à partir de la biopsie et du scanner abdomino-pelvien. Le traitement a consisté en une irradiation palliative à la dose de 30Gy. L’évolution a été marquée par le décès de la patiente. A travers ce cas clinique on a démontré que le muscle peut être touché par les métastases d'un carcinome épidermoïde du col utérin et que leur pronostic reste en général péjoratif.

## Introduction

Les métastases musculaires sont rares. La lésion primitive est le plus souvent un carcinome d'origine pulmonaire. Tous les muscles du corps peuvent être atteints mais il semble exister une prédominance des muscles psoas et para vertébraux.

## Patient et observation

Il s'agit d'une patiente âgée de 65 ans, sans antécédents pathologiques particuliers qui a présenté il y a 10 mois des métrorragies de moyenne abondance. L'examen initial trouve une tumeur du col utérin stade IIIB selon la classification de la FIGO (International Federation of Gynecology and Obstetrics). La biopsie du col utérin a objectivé un carcinome épidermoïde moyennement différencié peu kératinisant ulcéré et infiltrant. La tomodensitométrie (TDM) abdomino-pelvienne montre un processus tumoral cervico-isthmique respectant la vessie et le rectum sans adénopathies pelviennes ni lomboaortiques. Le reste des structures est sans particularités. Une radio-chimiothérapie concomitante suivie d'une curiethérapie utéro vaginale a été préconisées, cependant la patiente a abandonné le traitement après la 15ème fraction de radiothérapie.

**Figure 1:**
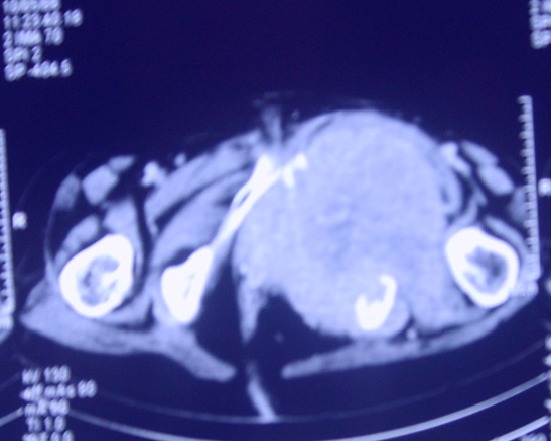
Coupe scannographique axiale montrant un processus tumoral lysant le corps du pubis et infiltrant les muscles de la cuisse gauche

**Figure 2:**
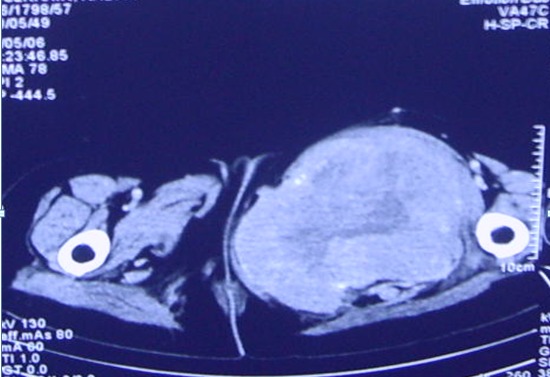
Coupe scannographique axiale montrant une masse musculaire hétérogène et nécrosée

**Figure 3:**
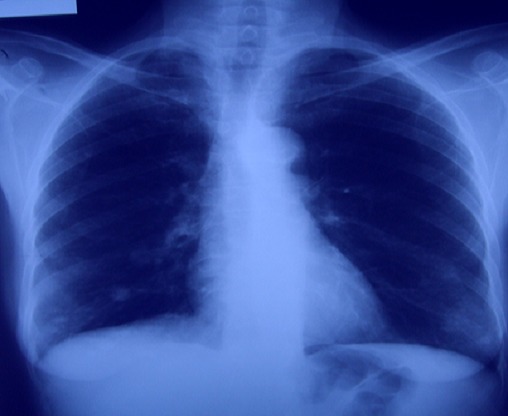
Radiographie pulmonaire de face objectivant des opacités pulmonaires bilatérales d'allure secondaire

Trois mois après; elle a présenté un nodule au niveau de la cuisse gauche augmentant progressivement de volume, l'examen trouve un blindage de la cuisse gauche avec un blindage pelvien aux touchers rectal et vaginal. La biopsie de la cuisse gauche a objectivé qu'il s'agit d'une métastase musculaire d'un carcinome épidermoïde peu différencié dont l'origine cervicale est tout à fait compatible. la TDM abdomino-pelvienne montre un volumineux processus expansif hypodense, hétérogène comportant des zones de nécrose et des images de débris osseux développés de part et d'autre du corps du pubis infiltrant le muscle obturateur interne et s’étendant dans le pelvis et au niveau de la région périnéale. Cette lésion infiltre les muscles de la loge interne de la cuisse avec lyse du corps du pubis et de la cavité cotyloïde (Figure 1, Figure 2), avec une lésion hypodense au niveau hépatique correspondant à une lésion secondaire. A la radiographie pulmonaire on note des multiples métastases pulmonaires bilatérales (Figure 3).

Le traitement a consisté en une irradiation palliative du pelvis et de la cuisse gauche à la dose totale de 30Gy en 10 fractions et sur 14 jours Un mois après la fin de la radiothérapie, la patiente est devenue très altérée sur le plan général, son statut de performance (PS) était à 3, elle est décédée suite à ses métastases.

## Discussion

Bien que plus de 45% de la masse corporelle soit constituée par le muscle squelettique, ce dernier est rarement le siège des métastases [1, 2]. Plusieurs hypothèses ont été évoquées afin d'expliquer la rareté de ces lésions [3, 4] : le muscle squelettique strié échappe au trajet des voies de dissémination cancéreuse essentielle, il faut donc évoquer une voie artérielle ; la résistance du muscle strié par la production d'acide lactique qui permet d’éviter l'anoxie cellulaire qui favorise le développement des cellules tumorale ; le muscle normal est capable de produire une forte réaction immunitaire qui empêcherait la fixation de cellules tumorales.

Les tumeurs à fort potentiel métastatique musculaire sont par ordre de fréquence : les carcinomes d'origine diverse (mammaire, bronchique, colique, gastrique), les lymphomes et les leucémies [1]. Le néoplasme d'origine gynécologique est également possible. Cliniquement les métastases musculaires peuvent être révélatrices d'un cancer non connu ou apparaître au cours de l’évolution d'un cancer connu après un délai variable, la douleur étant souvent le symptôme révélateur. Radiologiquement, aucun critère n'est spécifique [5]. La TDM peut objectiver un élargissement du corps musculaire par une lésion bien limitée hypodense avec des zones centrales de nécrose et une prise de contraste périphérique du tissu tumoral après injection du produit de contraste. L'imagerie par résonance magnétique (IRM) objectivera une lésion hypo- ou iso-intense par rapport au muscle sain en séquence pondérée en T1, les zones de nécrose centrales appariassent en hyper signal en séquences de pondération T2 [6, 7].

Toutefois, à défaut de confirmer la nature métastatique de la lésion, une ponction biopsie à l'aiguille de la masse permet de confirmer sa nature [8]. Le traitement des métastases musculaires n'est pas codifié. Trois moyens peuvent être utilisés, la chirurgie, la radiothérapie et la chimiothérapie [8, 9]. En cas des métastases musculaires douloureuses et survenant dans le cadre d'une maladie multi métastatique, une radiothérapie à visée antalgique, associée ou pas à une chimiothérapie, peut être indiquée tout en tenant compte du type de la maladie primitive, des autres organes touchés et de l’âge du patient [10]. Le pronostic des métastases musculaires est en général péjoratif, il est en fait directement lié au pronostic de la lésion primitive [8, 10].

## Conclusion

Les métastases musculaires sont rares. Elles peuvent révéler un cancer inconnu ou apparaître au cours de l’évolution d'un cancer connu. Aucun critère radiologique n'est spécifique. Le traitement de ces lésions n'est pas codifié et doit tenir compte de l’état du malade et de la lésion primitive.
